# Corrected partial anomalous pulmonary vein connection associated with lung resection: a case report

**DOI:** 10.1186/s40792-024-02070-x

**Published:** 2024-11-21

**Authors:** Kosuke Nakata, Jun Takaki, Toshihiro Fukui

**Affiliations:** Department of Cardiovascular Surgery, Kumamoto University Hospital, 1-1-1, Honjo, Chuo-Ku, Kumamoto 860-8556 Japan

**Keywords:** Lung cancer, Partial anomalous pulmonary venous connection, Revascularization

## Abstract

**Introduction:**

Partial anomalous pulmonary venous connection (PAPVC) is a potential cause of right-sided heart failure. Notably, a risk of sudden circulatory failure exists during lung surgery. Only a few reports of PAPVC complicated by lung cancer requiring lobectomy exist. Here, we report a case of left lower lung lobectomy complicated by an anomalous connection of the left upper pulmonary vein requiring revascularization.

**Case presentation:**

A 66-year-old man was found with an abnormal mass shadow in the left lower lung field on chest radiography. Bronchoscopy revealed lung adenocarcinoma. Preoperative contrast-enhanced computed tomography showed that the left upper pulmonary vein did not perfuse the left atrium but was connected to the left brachiocephalic vein. Preoperative transthoracic echocardiography revealed right atrial and ventricular enlargement. The clinical diagnosis was stage IB (T2aN0M0). We decided to perform a left lower lobectomy and correct the PAPVC to maintain oxygenation after the lobectomy. The PAPVC was ligated, and the stump of the left upper pulmonary vein was anastomosed to the left atrial appendage without cardiopulmonary bypass. Postoperative contrast-enhanced computed tomography revealed intact reconstructed vessels. Postoperative transthoracic echocardiography showed no right ventricular overload. The patient’s postoperative clinical course following the surgical procedure was uneventful. Furthermore, the patient was followed up without any complications.

**Conclusions:**

This rare case of successful surgical correction can inform clinicians of similar cases.

## Introduction

A partial anomalous pulmonary venous connection (PAPVC) is a congenital vessel anomaly. One or more pulmonary veins connect directly to the right atrium or indirectly flow through the systemic vein [1]. However, only a few reports of PAPVC complicated by lung cancer requiring lobectomy exist. The PAPVC is ligated if it is located in the pulmonary lobe designated for resection. However, determining whether revascularization should be performed if the lesion is located in a preserved lobe is necessary. Here, we report a case of left lower lung lobectomy complicated by an anomalous connection of the left upper pulmonary vein (LUPV) that required revascularization.

## Case presentation

**Fig. 1:**
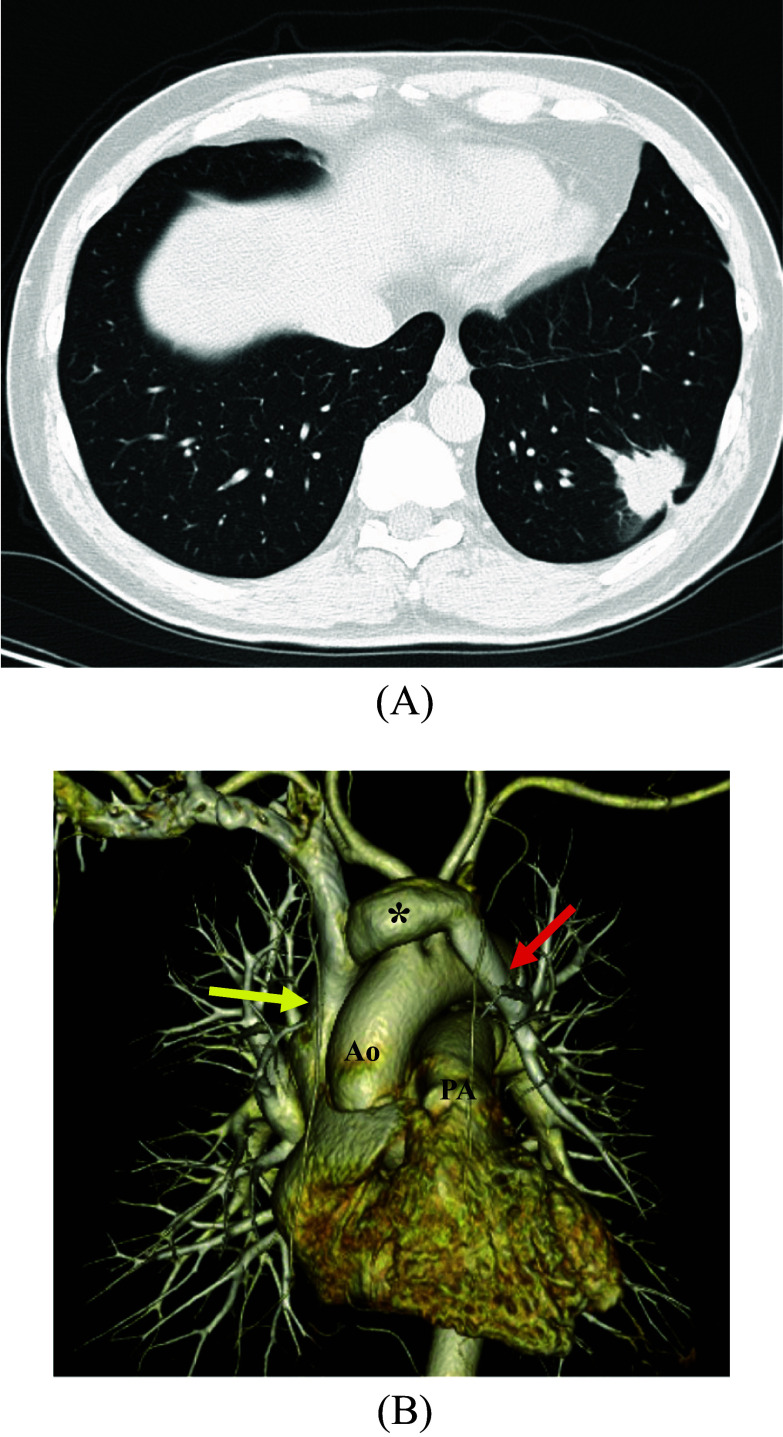
Preoperative contrast-enhanced computed tomography. **A** Mass with a specula was observed in the left lower lobe. Its border was irregular, and its diameter was ~ 39 mm. **B** Left upper pulmonary vein (red arrow: LUPV) drained into the superior vena cava (yellow arrow) via the left brachiocephalic vein (asterisk) (*Ao* aorta, *PA* pulmonary artery)

A 66-year-old man was found with an abnormal mass shadow in the left lower lung field on chest radiography. Bronchoscopy confirmed lung adenocarcinoma. Subsequently, the patient was referred to our hospital for surgical treatment. He was asymptomatic. On physical examination, the patient’s percutaneous oxygen saturation was 98% in room air, and his respiratory rate was 16/min. Pulmonary function tests showed the forced expiratory volume percent in 1 s was 70.57%, and the percent vital capacity was 101.1%. Preoperative contrast-enhanced computed tomography (CECT) revealed an ~ 39 mm mass in the left lower lobe (Fig. 1A). The LUPV did not perfuse the left atrium but was connected to the left brachiocephalic vein (Fig. 1B). Preoperative transthoracic echocardiography showed a left ventricular ejection fraction of 65.3%. No asynergies or atrial septal defects were found. The right atrium and ventricle were enlarged, and the clinical diagnosis was stage IB (T2aN0M0). We decided to perform a left lower lobectomy and correct the PAPVC to maintain oxygenation after the lobectomy.

**Fig. 2:**
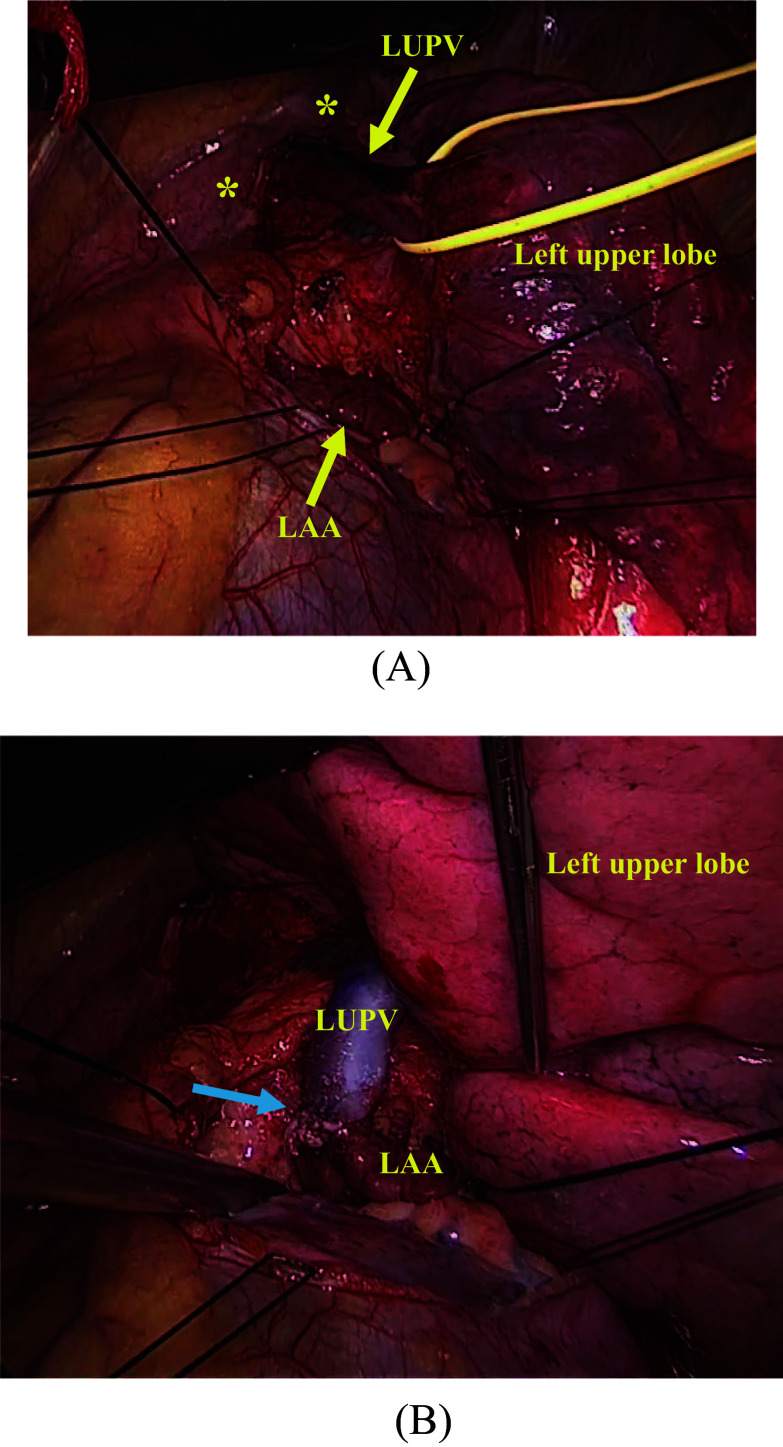
Intraoperative findings. **A** Left brachiocephalic vein was noted behind the pleura (asterisk). The left upper pulmonary vein (LUPV) connected to the left brachiocephalic vein. No pulmonary veins were entering the left atrium except for the left lower pulmonary vein. We marked the antero-wall of the LUPV to prevent kinking. **B** LUPV was anastomosed to the left atrial appendage. The blue arrow indicates the anastomotic site

First, the patient underwent video-assisted left lower lobectomy with lymphadenectomy through left posterolateral thoracotomy in the fifth intercostal space under one-lung ventilation. Subsequently, the abnormal LUPV was corrected. Intraoperatively, the LUPV drained into the left brachiocephalic vein and was not connected to the left atrium. The LUPV was peeled to the confluence of the brachiocephalic vein to ensure it was sufficiently long. In the same view, the left side pericardium was incised, and the left atrium appeared. We confirmed that the LUPV could be anastomosed to the left atrial appendage and was not kinked during lung inflation. After 80 units/kg of heparin was injected, the junction between the LUPV and the left brachiocephalic vein was ligated using a Powered ECHELON FLEX (ETHICON, NJ, USA). The left atrium appendage was partially clamped. Subsequently, the LUPV stump was anastomosed to the left atrial appendage using a 6-0 PROLENE (ETHICON, NJ, USA) without cardiopulmonary bypass (Fig. 2). Postoperatively, we initiated anticoagulation with warfarin and aspirin to prevent thrombosis at the anastomotic site.

**Fig. 3:**
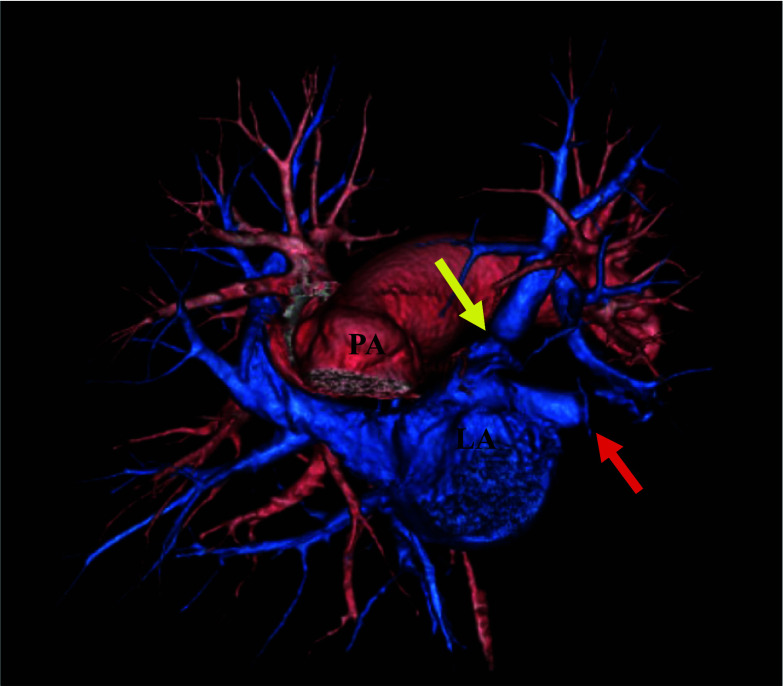
Postoperative contrast-enhanced computed tomography. The anastomotic area was intact and patent (yellow arrow). The red arrow indicates the stump of the left lower pulmonary vein

Postoperative CECT revealed intact reconstructed vessels (Fig. 3). Postoperative transthoracic echocardiography revealed no right ventricular overload.

The clinical course following the surgical procedure was uneventful, and the patient was discharged 8 days postoperatively. Additionally, the patient was followed up without any complications.

## Discussion

PAPVC is a rare congenital vascular anomaly; its frequency is estimated at 0.4–0.7% in postmortem examinations [2]. Notably, PAPVCs are usually detected in the right lung, with only 10% of anomalies occurring in the left lung [2]. Furthermore, 80% of PAPVC cases are complicated by atrial septal defects [3]. PAPVCs without atrial septal defects are relatively rare, as in our case. Specifically, one or more pulmonary veins failed to connect to the left atrium, resulting in a left-to-right shunt. The more the left-to-right shunt increases, the more severe the symptoms [4]. However, PAPVC may remain undiagnosed until adulthood because only a few clinical symptoms typically manifest [2]. Importantly, PAPVC is found in 0.1% of the adults examined using computed tomography [5]. Many reports have shown that older patients with PAPVC have an increased right ventricular strain and symptoms of right heart failure [2].

In the present case, we decided to perform a left lower lobectomy for radical resection. PAPVC was diagnosed incidentally during preoperative examinations.

A pulmonary-to-systemic flow ratio (Qp/Qs) of greater than 1.5 is one of the surgical indications of PAPVC [6]. However, the surgical indications for PAPVC have not yet been defined.

Blood from the PAPVC was perfused into the left brachiocephalic vein. Therefore, after lobectomy, we speculated that the hemodynamics would be similar to those found in left pneumonectomy due to blood flow from the PAPVC. We considered that the Qp/Qs increased and oxygenation decreased because the left-to-right shunt flow increased postoperatively.

Furthermore, we presumed that the patient had right heart overload because the preoperative transthoracic echocardiography showed right ventricular dilatation. ElBardissi et al. reported a patient who died of right heart failure due to uncorrected PAPVC after a lung cancer procedure [7]. Moreover, right-sided heart overload can cause right-sided heart failure in the long term. Therefore, we decided to correct the PAPVC.

However, the operative procedure has not yet been established. Five cases of reconstructive PAPVC were associated with lung resection (Table 1) [8–12]. If the PAPVC was located in the same lung lobe to be resected, we identified an abnormal vessel and ligated it. Otherwise, careful measures should be taken. PAPVC on the right side necessitates intra-atrial flow diversion with cardiopulmonary bypass, employing techniques such as the Warden procedure or the double-decker method [3, 12]. In contrast, the left PAPVC could be reconstructed without cardiopulmonary bypass. In the present case, the PAPVC was anastomosed to the left atrial appendage for anatomical reasons. Takei et al. anastomosed an anomalous vein to the stump of a pulmonary vein connected to a resected lobe [8].

**Table 1:** Reconstructive cases of partial anomalous pulmonary vein connection associated with lung resection

Year	Author	Age	Gender	Tumor location	Resection	PAPVC source	PAPVC drain	Anastomotic site
2002	Takei et al.	58	Female	LLL	Lobectomy	LUPV	LBCV	LLPV
2005	Sakurai et al.	48	Male	LUL	Pneumonectomy	RUPV	SVC	Intra-atrial flow diversion
2006	Smith et al.	45	Female	LLL	Lobectomy	LUPV	LBCV	LAA
2017	Kawagoe et al.	74	Female	LLL	Lobectomy	LUPV	LBCV	LLPV
2020	Fukumoto et al.	66	Female	LUL	Lobectomy	RUPV	SVC	Double-decker method
2024	Present case	66	Male	LLL	Lobectomy	LUPV	LBCV	LAA

*LUL* left upper lobe, *LLL* left lower lobe, *LUPV* left upper pulmonary vein, *LLPV* left lower pulmonary vein, *RUPV* right upper pulmonary vein, *LBCV* left brachiocephalic vein, *SVC* superior vena cava, *LAA* left atrial appendage

Postoperatively, anticoagulation therapy was initiated to prevent thrombogenesis at the anastomotic site. The target prothrombin time-international normalized ratio was set at 2.0. Transthoracic echocardiography performed in the second postoperative month revealed no right ventricular overload. Therefore, warfarin was discontinued because the reconstructed vessel was not obstructed, and aspirin was continued.

The patient’s postoperative course was uneventful. This rare case of successful surgical treatment of PAPVC with lung cancer in different lobes can guide clinicians in managing similar cases.

## Conclusions

PAPVC is a potential cause of right-sided heart failure. In particular, a risk of sudden circulatory failure exists during lung surgery. Therefore, the indications for surgical correction must be carefully determined. This rare case of successful surgical correction of PAPVC with lobectomy can inform clinicians in managing similar cases.

## Data Availability

None.

## Abbreviations

CECT: Contrast-enhanced computed tomography
LUPV: Left upper pulmonary vein
PAPVC: Partial anomalous pulmonary venous connection
Qp/Qs: Pulmonary-to-systemic flow ratio

## References

[1] Healey JE Jr. An anatomic survey of anomalous pulmonary veins: their clinical significance. J Thorac Surg. 1952;23:433–44. 10.1016/S0096-5588(20)31117-X. 14928263

[2] ElBardissi AW, Dearani JA, Suri RM, Danielson GK. Left-sided partial anomalous pulmonary venous connections. Ann Thorac Surg. 2008;85:1007–14. 10.1016/j.athoracsur.2007.11.038. 18291189 10.1016/j.athoracsur.2007.11.038

[3] Said SM, Burkhart HM, Schaff HV, Cetta F, Phillips SD, Barnes RD, et al. Single-patch, 2-patch, and caval division techniques for repair of partial anomalous pulmonary venous connections: does it matter? J Thorac Cardiovasc Surg. 2012;143:896–903. 10.1016/j.jtcvs.2011.09.074. 22325328 10.1016/j.jtcvs.2011.09.074

[4] Kawasaki H, Oshiro Y, Taira N, Furugen T, Ichi T, Yohena T, et al. Partial anomalous pulmonary venous connection coexisting with lung cancer: a case report and review of relevant cases from the literature. Ann Thorac Cardiovasc Surg. 2017;23:31–5. 10.5761/atcs.cr.16-00110. 27321230 10.5761/atcs.cr.16-00110 PMC5347485

[5] Ho M-L, Bhalla S, Bierhals A, Gutierrez F. MDCT of partial anomalous pulmonary venous return (PAPVR) in adults. J Thorac Imaging. 2009;24:89–95. 10.1097/RTI.0b013e318194c942. 19465830 10.1097/RTI.0b013e318194c942

[6] Singhal K, Newton AD, Corbett C, Predina JD. Management of partial anomalous pulmonary venous connections in patients requiring pulmonary resection: a case report and systematic review. J Thorac Dis. 2017;9:5434–9. 10.21037/jtd.2017.11.68. 29312754 10.21037/jtd.2017.11.68 PMC5757009

[7] Black MD, Shamji FM, Goldstein W, Sachs HJ. Pulmonary resection and contralateral anomalous venous drainage: a lethal combination. Ann Thorac Surg. 1992;53:689–91. 10.1016/0003-4975(92)90336-3. 1554284 10.1016/0003-4975(92)90336-3

[8] Takei H, Suzuki K, Asamura H, Kondo H, Tsuchiya R. Successful pulmonary resection of lung cancer in a patient with partial anomalous pulmonary venous connection: report of a case. Surg Today. 2002;32:899–901. 10.1007/s005950200176. 12376789 10.1007/s005950200176

[9] Sakurai H, Kondo H, Sekiguchi A, Naruse Y, Makuuchi H, Suzuki K, et al. Left pneumonectomy for lung cancer after correction of contralateral partial anomalous pulmonary venous return. Ann Thorac Surg. 2005;79:1778–80. 10.1016/j.athoracsur.2003.10.092. 15854983 10.1016/j.athoracsur.2003.10.092

[10] Smith RL II, Zorn GL III, Peeler BB, Jones DR. Combined bronchial sleeve resection and repair of partial anomalous pulmonary venous return. J Thorac Cardiovasc Surg. 2006;132:982–3. 10.1016/j.jtcvs.2006.05.062. 17000322 10.1016/j.jtcvs.2006.05.062

[11] Kawagoe I, Hayashida M, Nozumi Y, Banno T, Hirayama S, Suzuki K, et al. A combination of a partial anomalous pulmonary venous connection (PAPVC) and a lung tumor requiring pulmonary resection. J Cardiothorac Vasc Anesth. 2017;31:274–8. 10.1053/j.jvca.2016.06.011. 27645823 10.1053/j.jvca.2016.06.011

[12] Fukumoto K, Goto M, Ichikawa Y, Kawasumi Y, Uchiyama M, Maekawa A, et al. Lobectomy with bronchoplasty and pulmonary arterial angioplasty for lung cancer after correction of contralateral partial anomalous pulmonary venous connection. Surg Case Rep. 2020;6:310. 10.1186/s40792-020-01083-6. 33284359 10.1186/s40792-020-01083-6 PMC7721844

